# Neurokinin Antagonists to Treat Vasomotor Symptoms—Possible Implications for Long-Term Health and Disease

**DOI:** 10.3390/jcm14196852

**Published:** 2025-09-27

**Authors:** Angelo Cagnacci, Anjeza Xholli, Marta Fiamberti, Ambrogio Pietro Londero

**Affiliations:** 1 Academic Unit of Obstetrics and Gynecology, IRCCS-San Martino Hospital, 16136 Genoa, Italymartafiamberti@gmail.com (M.F.); 2 Department of Neuroscience, Obstetrics and Gynecology Unit, Rehabilitation, Ophthalmology, Genetics and Maternal and Pediatric Sciences, University of Genova, 16148 Genoa, Italy; 3 Obstetrics and Gynecology Unit, IRCCS Istituto Giannina Gaslini, 16147 Genoa, Italy

**Keywords:** neurokinin antagonist, vasomotor symptoms, menopause, elinzanetant, fezolinetant, cardiovascular disease, osteoporosis, sleep, depression, mood, metabolic syndrome, oxidative stress, cortisol

## Abstract

In women in post-menopause, the presence of severe vasomotor symptoms is associated with sleep disorders and a depressive mood. Vasomotor symptoms, sleep disorders, and a depressive mood are all related to an increased risk of cardiovascular events and bone fractures. The association is still elusive, but some mechanisms may sustain a hypothetical causal relation. During flush, the heart rate increases, augmenting blood turbulence and possibly posing a risk for endothelial damage. Altered sleep is associated with a reduced nocturnal blood pressure decline, which represents a risk factor for cardiovascular disease. Cortisol levels rise during each flush but also following sleep deprivation or in individuals with depression. Increased cortisol was found in women with menopausal symptoms and can induce insulin resistance, metabolic syndrome, cardiovascular disease, and bone demineralization. An elevated oxidative state is associated with vasomotor symptoms, sleep disturbances, and depression and increases the risk of cardiovascular events and osteoporosis. The use of non-hormonal remedies for symptom management leads to a decrease in blood pressure and a reduction of 24 h urinary cortisol, contingent upon the extent of symptom alleviation. Recent evidence indicates that fezolinetant, a neurokinin-3 receptor antagonist and elinzanetant, a neurokinin-1-3 receptor antagonist, diminish the frequency and severity of vasomotor symptoms. As the secondary endpoint of these studies, some amelioration of patients reported that sleep disturbance was observed during fezolinetant and more consistently during elinzanetant. Some improvement in the quality of life and depressive mood were also observed during elinzanetant. The causal relation of symptoms with cortisol levels and oxidative stress, and the reduction in cortisol and blood pressure by symptom improvements, support the possibility that neurokinin antagonists may decrease those factors linking menopausal symptoms with cardiovascular disease and osteoporosis. Dedicated studies are needed to test the hypothetical possibility that neurokinin receptor antagonists contribute to reduce the long-term burden of cardiovascular disease and osteoporosis of symptomatic women in post-menopause unwilling or with contraindication to the use of menopause hormone therapy.

## 1. Introduction

Nearly twenty years ago, two articles first documented the potential link between menopausal vasomotor symptoms and risk factors for cardiovascular disease. An article indicated that women experiencing vasomotor symptoms exhibited a higher body mass index, cholesterol levels, and both systolic and diastolic blood pressure compared to asymptomatic women [1]. Another study indicated that women experiencing vasomotor symptoms exhibited altered endothelial function, characterized by diminished flow-mediated endothelium-dependent vasodilation, alongside advanced calcification of the aortic and coronary arteries [2]. In subsequent years, an increasing volume of data was gathered, which reinforced, albeit inconsistently [3,4,5], the link between vasomotor symptoms and cardiovascular risk factors [6,7,8,9]. Data indicating that the prevalence of cardiovascular events is elevated in women experiencing vasomotor symptoms [10,11,12,13,14,15,16,17] ultimately confirmed the association between the presence of symptoms and the subsequent manifestation of cardiovascular disease.

During the same period, other researchers investigated the relationship between vasomotor symptoms and postmenopausal osteoporosis. The authors presented compelling evidence of heightened bone turnover leading to bone loss in symptomatic women during the menopausal transition [18], as well as of diminished bone mineral density in symptomatic versus asymptomatic women in post-menopause [19,20]. They also presented evidence indicating a higher incidence of bone fractures among symptomatic women [21].

The prospect that symptoms are associated with a lower health status was further reinforced by research indicating that symptoms may correlate with impaired short-term memory [22,23]. Furthermore, nighttime vasomotor symptoms are associated with the onset of neurodegenerative diseases, as evidenced by the presence of brain white matter hyperintensities [24], likely via alterations in cerebral blood flow [25].

Associations between discomfort induced by menopause symptoms and other peripheral disturbances were also reported. Increased scores of menopausal symptoms assessed by Greene’s climacteric scale correlated with a higher prevalence of pelvic organ prolapse overall and specifically of bladder prolapse [26]. Higher scores of Greene’s climacteric scale were observed in women experiencing urinary incontinence [27], and the association between scale scores and stress, urge, and mixed urinary incontinence remained independent of the other risk factors considered [27].

This evidence suggests that symptomatic women exhibit a heightened sensitivity to hormone fluctuations in perimenopause and to hormone withdrawal in post-menopause. This increased sensitivity manifests across various levels, including the central neurotransmitters, cardiovascular system, bones, and body tissues. Accordingly, the presence of symptoms may serve as a criterion to identify women who are more likely to benefit in the long-term from menopause hormone therapy [28], even if this hypothesis has not been rigorously tested or validated.

Another aspect not fully considered is that symptoms, “per se”, may contribute to long-term health disturbances by inducing biological modifications that influence risk factors for cardiovascular disease or osteoporosis. In this case, remedies specifically designed to treat menopausal symptoms, such as neurokinin receptor antagonists, may uncover previously unknown capabilities that can potentially enhance long-term health outcomes in women in post-menopause.

## 2. The Burden of Menopausal Symptoms

Vasomotor symptoms represent the hallmark of menopausal symptoms. Their characteristics include rapid vasodilation and perspiration for heat loss, followed by horripilation and shivering to generate heat [29,30,31,32].

During the menopausal transition and post-menopause, women experience vasomotor symptoms with a different trajectory [33,34,35,36]. Some women may have minimal or no vasomotor symptoms, others may encounter them only before or only after menopause, and others both before and after the menopause. Research indicates that symptoms tend to persist longer when initiated in perimenopause and in Black women [33,35] and less in Asian women [33,35].

Women experiencing vasomotor symptoms exhibit a greater prevalence of sleep disturbances [37,38,39,40]. Individuals may encounter challenges in initiating sleep, experience frequent nighttime awakenings, or wake up earlier in the morning. A cause–effect relationship between nocturnal vasomotor symptoms and awakening has been reported [41], but the two may also represent a shared manifestation of neurotransmitter changes induced by hormonal fluctuations. For example, hypothalamic kisspeptin–neurokinin–dynorphin (KNDy) neurons release neurokinin B in the medial preoptic area of the hypothalamus, where it can influence the neurons of thermoregulation through neurokinin-3 receptors and the neurons of sleep via neurokinin-1 receptors [42,43]. Likely, vasomotor symptoms contribute to sleep disruption in conjunction with other changes brought about by the hormonal environment of peri- and post-menopause [32,38].

Mood alterations, depression, and anxiety are symptoms that, while not exclusive to menopause, are reported by women to intensify during the menopause transition [44,45,46,47,48]. The presence of vasomotor symptoms [49,50] and alterations of sleep [51,52,53] are correlated with a negative mood, and mechanisms supporting a causal link among them can be envisioned.

Menopause symptoms include also peripheral manifestations, like joint pain and genitourinary issues [47]. Symptoms of vaginal atrophy, such as vaginal dryness and dyspareunia, increase across the menopausal age [54], and contribute, along with the changed endocrine milieu, to a progressive reduction in sexuality [55]. Symptoms of vaginal atrophy also correlate with low urinary tract symptoms [56], and symptoms of menopause correlate with urinary incontinence of any type [27].

The associations between vasomotor symptoms and sleep problems, between sleep alterations and low mood, and between menopausal symptoms and urinary disturbances may suggest probable mechanistic links. These associations raise the possibility that the effective treatment of one symptom domain may exert beneficial effects across other symptom manifestations.

## 3. Associations Between Menopause Symptoms and Long-Term Health Consequences

### 3.1. Cardiovascular Disease

Early, premature, or surgical menopause is linked to a heightened risk of cardiovascular disease [57,58,59,60,61,62]. Women in post-menopause exhibit a greater cardiovascular risk compared to their premenopausal counterparts of a similar age [63].

The prevalence of metabolic syndrome, defined by its components of insulin resistance, diabetes, visceral adiposity, lipid abnormalities, elevated blood pressure, and chronic inflammation, rises in the years preceding menopause and continues in post-menopause [64,65,66].

Recent evidence over the past two decades suggests a correlation between the components of the metabolic syndrome and an increased frequency of vasomotor symptoms. Women experiencing vasomotor symptoms exhibit increased weight, visceral adiposity, and lipid abnormalities [1,6,8,9,67]. Additionally, they show heightened inflammation alongside oxidative stress [17,68,69,70], elevated blood pressure [1,2,71,72], although this is not universally confirmed [3], elevated glucose, insulin resistance, and diabetes [1,6,8,9,73,74]. Arterial damage is documented by a reduction in flow-mediated endothelium-dependent vasodilation [2,75], an increase in the intima–media thickness of the internal carotid artery [76], and an advancement of calcified atherosclerosis in the coronary and aortic arteries [77,78]. Consistently, the risk of cardiovascular disease and cardiovascular mortality is elevated [10,11,12,13,14,16,79].

Also, sleep disruption is linked to a heightened risk of metabolic syndrome [80], the nocturnal elevation of blood pressure, and a higher risk of hypertension [81,82] and cardiovascular disease [40,83,84]. Similarly, depressive symptoms are causally linked to the metabolic syndrome [85] and associated with an increased risk of cardiovascular disease [53,86].

The studies using scales that measure the disturbance of menopausal symptoms still demonstrated an association with cardiovascular risk factors. Elevated scores of the Greene’s climacteric scale correlated with various health concerns, such as a higher body mass index and increased waist circumference [87,88], hypertension [89], and lipid abnormalities leading to an increased predicted 10-year risk of cardiovascular events [87,88,90]. Elevated scale scores correlated with increased glucose levels, insulin resistance [87,88], an increased prevalence of metabolic syndrome and diabetes [65], and heightened oxidative stress, along with an increased resistance to arterial blood flow [91]. Menopausal symptoms, in general, were also correlated with an increased risk of cardiovascular disease and all-cause mortality [92].

Therefore, data based on vasomotor symptoms or on the burden of menopausal symptoms demonstrate a clear association with cardiovascular disease. This association remains unclear, and it is crucial to investigate whether intermediate biological mechanisms could elucidate a causal relationship between symptoms and cardiovascular disease.

### 3.2. Osteoporosis

Women experiencing early, premature, or surgical menopause exhibit earlier and accelerated [93] bone mineral loss, leading to a decrease in bone mineral density at the time of natural menopause [60,61,93,94,95,96]. A higher prevalence of bone fractures is also observed in this population [97]. Bone turnover and loss increase significantly during the menopausal transition, particularly in the late perimenopause [98].

Women experiencing vasomotor symptoms exhibit an increase in these accelerations [18], and in contrast to asymptomatic or minimally symptomatic women, in post-menopause, they demonstrate a lower bone mineral density [19,20] and a heightened risk of bone fractures [21].

Alterations in sleep are associated with negative effects on bone health [99]. In women in post-menopause, sleep alterations are linked to an imbalance between bone formation and absorption, resulting in a decreased bone mineral density [100,101] and, with other risk factors, they contribute to the increase in the risk of bone fractures [102].

There is no clear data on depressive moods, but major depressive episodes are linked to changes in bone turnover, inducing a decrease in bone mineral density [103,104,105,106], and in post-menopause, to an increase the risk of osteoporosis [107].

Therefore, the risk of osteoporosis is also associated with the presence of menopause symptoms, but its causal link and its origin remain unclear.

## 4. The Hypothetical Link Between Symptoms and Long-Term Health Consequences

**Figure 1:**
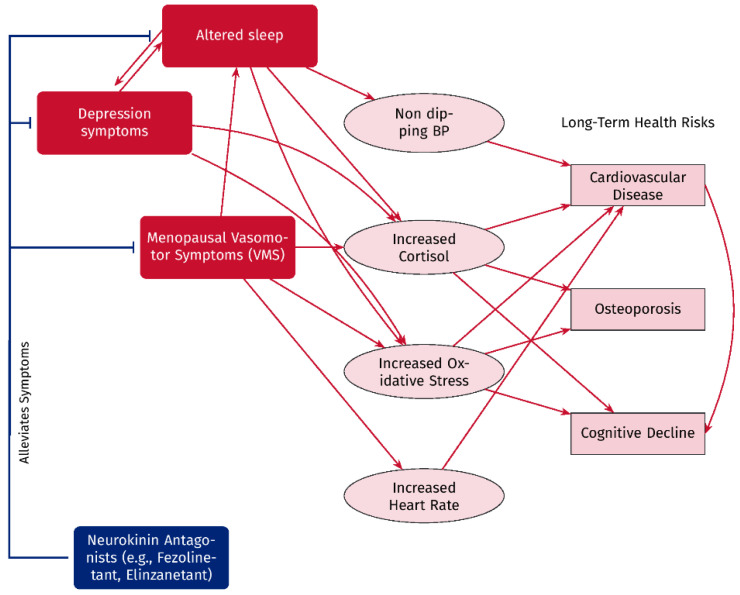
Pathway illustrating the hypothetical connection between menopausal vasomotor symptoms (VMSs), altered sleep, and depressive symptoms, and their hypothetical association with long-term health risks. VMSs and depression affect sleep patterns, which in turn influence the symptoms of depression in a bidirectional manner. These factors induce biological changes such as non-dipping blood pressure, elevated cortisol levels, oxidative stress, and increased heart rate, all of which correlate with increased risks for cardiovascular disease, osteoporosis, and cognitive decline. Neurokinin antagonists, such as fezolinetant and elinzanetant, may mitigate vasomotor symptoms, depressive symptoms, and sleep disturbances, potentially decreasing the associated biological changes and the long-term health risks.

Understanding the biological modifications associated with vasomotor symptoms is crucial for elucidating the possible potential relationships between these symptoms and the risk factors for cardiovascular disease and osteoporosis (Table 1 and Figure 1). Additionally, exploring the biological mechanisms linking disrupted sleep or depressive symptoms to cardiovascular and bone risk factors is crucial.

### 4.1. Vascular Modifications

Hot flushes are characterized by an abrupt peripheral vasodilation that facilitates heat dissipation [32]. During a flush, there is a reduction in cardiac vagal control [108], accompanied by the production of a calcitonin gene-related peptide, a vasodilatory peptide [109]. The reduction in peripheral vascular resistance correlates with an increase of approximately 15 beats per minute in the heart rate [29]. An increase in the heart rate elevates blood turbulence at arterial bifurcations, promoting endothelial damage and atherosclerosis [110,111,112], and represents an independent risk factor for both cardiovascular and overall mortality [113]. In animal models, the pharmacological reduction in the heart rate decreases oxidative stress, improves endothelial function, and prevents atherosclerosis [110]. Sleep alterations enhance the risk of hypertension, particularly of nocturnal non-dipping [81,82], which also represents an independent risk factor for cardiovascular disease [114,115,116].

### 4.2. Cortisol Modifications

Elevated cortisol levels have been observed during the menopausal transition [117] and in women after menopause, especially around lunchtime and in the early part of the night [118].

Menopausal symptoms may increase cortisol levels. Each flush is accompanied by the simultaneous secretion of pituitary hormones, including the luteinizing hormone (LH) [119,120] and adrenocorticotropic hormone (ACTH), along with various adrenal steroids including cortisol [120,121]. The increase in cortisol levels, ranging from 50% to 80% above the baseline, persists for a duration of 20 to 35 min [120,121] and may significantly contribute to the 24 h hormone exposure when flush frequency is markedly elevated. Research indicates that cortisol levels, assessed in hair or saliva, are elevated in women experiencing vasomotor symptoms [69,122,123].

An increase in cortisol may also occur alongside other menopausal symptoms. Changes in sleep architecture are associated with elevated cortisol, and sleep deprivation elevates cortisol [124,125,126,127]. Cortisol is also elevated in individuals experiencing depressive symptoms [122,128,129]. Consistently, women with an increased disturbance from menopausal symptoms, evaluated by the Greene’s climacteric scale, showed an elevated 24 h urinary cortisol [87]. Scale scores exhibited a linear relationship with urinary cortisol, with higher scores being associated with higher 24 h urinary levels [87].

An elevation of cortisol induces insulin resistance and leads to the development of the metabolic syndrome [130,131,132,133], which includes major risk factors for cardiovascular disease. Indeed, the literature data indicate that elevated cortisol is associated with an increased cardiovascular mortality following myocardial infarction [134]. An increased cardiovascular mortality associated with high cortisol levels was also documented in depressed [135] and in aged individuals [136,137]. Older adults exhibiting 24 h urinary cortisol levels in the middle and upper thirds of the distribution demonstrate cardiovascular mortality rates that are 2.9 and 5.0 times higher, respectively, than those in the first third of the distribution [136]. Women experiencing symptoms in post-menopause exhibit 24 h urinary cortisol levels that align with the middle and upper thirds of that reported distribution [87,136].

Cortisol may also play a role in the pathogenesis of osteoporosis. Elevated cortisol correlates with an accelerated bone loss [138], a decreased bone mineral density [139,140], and an increased incidence of bone fractures [139,140,141].

An increase in cortisol aligns with various other symptoms associated with menopause. Elevated cortisol contributes to gut dysbiosis, which promotes chronic low-grade inflammation and depression [129]. It also reduces sleep duration [142], impairs memory consolidation [143], hinders short-term memory recall [144], and may lead to mild cognitive impairment [145].

Thus, there is a lot of evidence implying that menopause symptoms are associated with an increase in cortisol and that cortisol elevation has the capability to interact and negatively modify all the risk factors for cardiovascular disease, osteoporosis, and also short- and long- term cognitive functions.

### 4.3. Oxidative Stress

Menopause appears to be associated with increased oxidative stress, presumably consequent to steroid hormone fluctuations and the estrogen withdrawal of post-menopause [68]. Women with vasomotor symptoms demonstrate heightened oxidative stress, presumably resulting from the increased metabolic rate associated with these symptoms [68,70]. Increased oxidative stress has also been documented in women with sleep disorders, particularly sleep apnea [146,147], and in association with depression [148,149,150]. Women exhibiting elevated scores of the Greene subscale for vasomotor symptoms demonstrated heightened oxidative stress, resulting from a significant decrease in antioxidant defenses [91]. Antioxidant values correlated with an increased resistance to arterial blood flow [91], a condition frequently associated with hypertension, diabetes, lipid abnormalities, cerebral atherosclerosis, and stroke [151]. Oxidative stress is linked to diminished endothelium-dependent vasodilation [152] and a heightened risk of cardiovascular disease [153,154,155,156].

In post-menopause, oxidative stress is also related to an increase in the markers of bone reabsorption [157], to a reduced bone mineral density [103,158,159], and to an increase in bone fractures [160].

## 5. Evidence from the Treatment of Menopause Symptoms

### 5.1. Menopause Hormone Therapy

Scientific societies and contemporary literature suggest that menopausal hormone therapy is indicated solely for symptomatic women due to its positive impact on symptoms and quality of life [161,162,163,164]. Menopausal hormone therapy exerts a generalized effect on the female body, exerting a positive effect on all menopausal symptoms, preserving bone mass and preventing bone fracture, improving women’s metabolism, atherosclerosis progression, and the genitourinary syndrome of menopause.

The Women’s Health Initiative (WHI) showed that the use of menopause hormone therapy may slightly decrease cardiovascular events when given within a decade since menopause but not later when it can become detrimental [165]. As a consequence, a theory was formulated that menopausal hormonal therapy should be given in a window of opportunity close to menopause when it is unlikely to cause harm, and it may even prevent cardiovascular events [166]. In older women with advanced atherosclerosis, the procoagulant effect of estrogens can be detrimental and cause an increased risk of cardiovascular accidents. Many clinicians argued that [166] the results of the WHI study cannot be applied to symptomatic women in the immediate post-menopause period because, by design, most enrolled women were of an older age and asymptomatic. A recent secondary sub-analysis of the WHI investigations evaluated whether the presence of vasomotor symptoms at baseline can change how the rate of cardiovascular events is modified using menopause hormone therapy. The intensity and frequency of vasomotor symptoms was not investigated, the categorization of night sweats was unclear, and women with reported severe symptoms, on a four-point scale from absent to severe, were not separately investigated from the other categories. The study showed no difference in cardiovascular events between symptomatic and asymptomatic treated women within 10 years since menopause [167]. In older women, menopause hormone therapy induced a greater risk of cardiovascular events in symptomatic than asymptomatic women, confirming a more compromised cardiovascular system in the former than in the latter [167].

Observational studies more appropriately represent the real-life use of menopausal hormone therapy, which is given to symptomatic women in a period very close to menopause. The bulk of the observational studies performed in these conditions indicate that hormone therapy decreases the risk of cardiovascular disease by approximately 50% [168]. A placebo-controlled study was purposedly performed with the primary endpoint of evaluating the modification of cardiovascular risks of symptomatic women in recent menopause. In accordance with the observational studies, the investigation showed that symptomatic women randomized to hormone therapy experienced a 50% reduction in events after 10 years [169].

The limited evidence suggesting an effect of menopause hormone therapy in reducing the cardiovascular risk in symptomatic more than asymptomatic women in recent menopause [168,169], and the age-related acceleration of cardiovascular diseases in symptomatic women [13,167], may support the possibility that symptom reduction may contribute to cardiovascular protection.

Bone loss is reduced by hormonal contraceptives in perimenopause [170,171] and by menopause hormone therapy after menopause, regardless of the route of its administration [172,173,174]. The WHI study provided the most robust data on fracture prevention, indicating that women randomized to hormone therapy experienced a 25% to 35% reduction in bone fractures [173]. The latter evidence indicates that the effect of menopause hormone therapy on bones is also significant in asymptomatic women. Whether symptomatic women experience potential additional benefits remains unexplored.

To note, menopause hormone therapy is capable of reducing all those intermediate factors that may connect symptoms to cardiovascular disease and osteoporosis, like cortisol [44,175,176], oxidative stress [68,70], and elevated nocturnal blood pressure [177,178].

### 5.2. Non-Hormonal Remedies

Non hormonal remedies can be used to alleviate symptoms when menopausal hormone therapy is not accepted or cannot be administered. Numerous non-hormonal interventions have been employed to alleviate vasomotor symptoms. Several herbal compounds and acupuncture have demonstrated a certain degree of efficacy among non-medical remedies [179,180,181].

Certain neuroactive drugs have demonstrated efficacy by enhancing the opioidergic (veralipride) [182,183], GABAergic (gabapentin), or serotonergic (SSRIs) signaling, as well as by directly diminishing the noradrenergic activity (clonidine) [31,32,184].

The possibility that symptom alleviation induces the modification of cortisol was investigated in a single randomized clinical trial. The study indicated that acupuncture alleviates climacteric symptoms to a degree like that of menopausal hormone therapy, as assessed by Greene’s climacteric scale and its vasomotor subscale, with both interventions showing superior efficacy compared to phytoestrogens [181]. In the same cohort, baseline Greene’s climacteric scale scores exhibited a linear relationship with 24 h urinary cortisol, further establishing a direct association between symptom severity and cortisol levels [179]. The reduction in the Greene’s scale score induced by treatment was linearly related to the decrease of 24 h urinary cortisol regardless of the therapy used, indicating that the reduction in symptom burden leads to a decline in cortisol [179]. Therefore, the effective management of menopausal symptoms may reduce prolonged cortisol exposure, potentially alleviating its recognized negative impacts on cardiovascular, metabolic, and neuropsychological health. The beneficial effect on cardiovascular risk factors of these modifications remains to be determined, but both acupuncture and phytoestrogens induced a decrease of approximately 6–8 mmHg of systolic and diastolic blood pressure from the baseline [185]. The impact on oxidative stress was not evaluated.

## 6. Effect of Neurokinin Antagonists

Neurokinin B has been recently identified as a significant neuropeptide in the pathogenesis of vasomotor symptoms [43]. Neurokinin B is synthesized by KNDy neurons situated in the arcuate nucleus, which project to the medial preoptic area of the hypothalamus. In post-menopause, KNDy neurons exhibit hypertrophy and an increase in neurokinin gene expression [43]. The release of neurokinin B in the medial preoptic area affects neurons of the thermoregulatory centers through neurokinin-3 receptors and causes peripheral vasodilation and heat loss [43]. A similar effect is exerted in women by the administration of neurokinin B [186]. In experimental animals, estrogen reduces vasomotor instability by decreasing the activity of KNDy neurons [187]. In women in post-menopause, the administration of selective neurokinin B receptor antagonists decreases both the frequency and the intensity of vasomotor symptoms [188,189,190,191,192,193]. Two 12-week, randomized, placebo-controlled clinical trials demonstrated that fezolinetant, a selective neurokinin-3 receptor antagonist, reduces both the frequency and severity of moderate to severe vasomotor symptoms [194,195]. Following a 12-week placebo-controlled phase, all patients who completed this period were eligible to participate in a subsequent 40-week active treatment extension. The treatment’s efficacy was sustained throughout the evaluation period [194,195]. Other clinical trials examined the efficacy of elinzanetant, a neurokinin-1-3 receptor antagonist, in treating moderate to severe vasomotor symptoms [196,197]. Two placebo-controlled studies demonstrated that after 12 weeks of treatment, elinzanetant significantly decreases the frequency and intensity of vasomotor symptoms [197]. Subsequently, all participants received elinzanetant for an extended period of 14 weeks. The clinical efficacy was maintained throughout the treatment period [197]. A recent study confirmed the efficacy of elinzanetant in breast cancer survivors on adjuvant hormone therapy with tamoxifen or aromatase inhibitors [198]. In this instance, elinzanetant demonstrated its efficacy vs. placebo during the initial 12 weeks that was conserved throughout the following 40 weeks of treatment.

**Figure 2:**
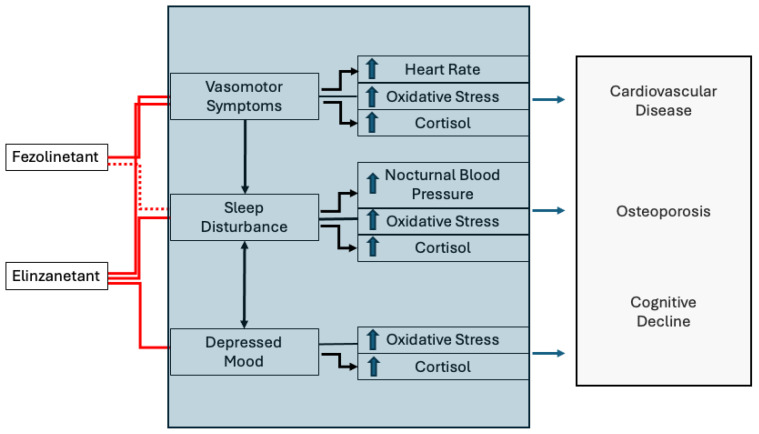
Hypothetical model linking the possible preventive effect of neurokinin antagonists on long-term health outcomes. The red lines indicate the demonstrated effects of fezolinetant and elinzanetant in reducing menopausal symptoms. The dotted red line indicates an uncertain direct effect of fezolinetant on sleep. The colored box reports the demonstrated association between symptoms and risk factors for long-term health consequences. The straight lines indicate associations without evidence of causality. The black arrows identify causal links. The blue arrows identify an increase in the identified function or activity. The hypothetical model suggests that neurokinin antagonists, by acting on symptoms, may reduce the output of the negative signals herein included in the colored box, leading to a reduction in long-term health consequences.

The effect of fezolinetant or elinzanetant exceeds the improvement of vasomotor symptoms (Figure 2). Secondary outcomes of the clinical studies with neurokinin receptor antagonists were modification from the baseline of the patient-reported outcomes measurement information system, the sleep disturbance SHORT Form 8b (PROMIS SD-SF-8b) total T score, with higher scores indicating more disturbed sleep. Women were not selected based on their seep quality, but women suffering from vasomotor symptoms exhibit a greater prevalence of sleep disturbances [37,38,39,40]. Although fezolinetant is reported to induce sleep disturbance in 3% of individuals [199], the studies showed that sleep quality slightly improved during its administration [194,195]. Sleep improvement appeared more consistent during elinzanetant [191,197,198,200]. These effects on sleep could be mediated by the complex relationship between vasomotor symptoms improvement and sleep; but, direct mechanisms, particularly for elinzanetant, can be anticipated. KNDy neurons of the arcuate nucleus project to the preoptic hypothalamus [43], releasing neurokinin B at neurons involved in sleep regulation [42]. The stimulation of the neurokinin-1 receptor through a selective agonist alters sleep patterns and induces insomnia [201]. Vice versa, the use of a selective neurokinin-1 receptor antagonist enhances sleep quality [202]. Elinzanetant is also a neurokin-1 receptor antagonist, and this property may explain its effect on sleep supposedly being superior to that of fezolinetant [191].

Studies have shown that fezolinetant also improves the health-related quality of life [203] and that elinzanetant also improves mood and working activity [204].

Accordingly, neurokinin receptor antagonists demonstrate effects that extend beyond their impact on vasomotor symptoms, contributing to improved sleep, mood, and overall quality of life. The investigation of these aspects is limited, but the available data suggests a potentially significant therapeutic effect, which may be pertinent when evaluating the long-term health consequences of symptom reduction in women in post-menopause. No study has evaluated modifications of cardiovascular risk factors during neurokinin antagonist administration. By contrast a recent investigation reported a secondary analysis on BMD changes during a 52-week elinzanteant administration [205]. In comparison to the placebo, elinzantenat improved the frequency and intensity of vasomotor symptoms and patient-referred sleep disturbances. The BMD decline of the femoral neck, hip, and lumbar spine was −0.6%, −1.2%, and −1.4%, during placebo and −0%, −0.7%, and −0.6% during elinzanetant. The short-term exposure to the treatment (1 yr.) was inappropriate to document a statistically significant effect, but the decline during active treatment was almost half that observed during the placebo. Similar considerations can be made when considering the BMD decline reported in a cohort of untreated multiethnic women in post-menopause whose yearly BMD declines range from −1.4% to −2.0% at the hip and spine, respectively [98].

## 7. Future Prospective

Observational studies indicate a correlation between the prevalence of vasomotor symptoms and, more broadly, of menopause symptoms, with long-term health outcomes. Limited interventional studies indicate that symptom improvement may have an influence on the risk factors leading to the long-term health outcomes, including cardiovascular disease and osteoporosis [179,185]. Selective neurokinin antagonists improve menopause symptoms, and their availability represents a distinct opportunity to test this possibility. These drugs, specifically designed to alleviate vasomotor symptoms [189,190,191,192], also demonstrate a beneficial impact on sleep and depressive moods via direct or indirect mechanisms.

Future studies need to address whether these drugs can reduce the 24 h exposure to cortisol and oxidative stress. The administration of a neurokinin-1 receptor antagonist, such as elinzanetant, may reduce cortisol also via direct effects exerted on the adrenal neurokinin-1 receptors [206,207].

Evidence on cardiovascular events requires long-term exposure and large cohorts of treated women. Indirect surrogate parameters can be studied more quickly, like the modification of blood pressure, particularly nighttime blood pressure, of glucose-insulin and lipoprotein metabolism. Studies dealing with intermediate surrogate markers of cardiovascular events like endothelial function and atherosclerosis progression may also contribute to the knowledge on the field.

Short-term studies may evaluate modifications of markers of bone turnover induced by treatment. Yet studies with a sufficient time of exposure of at least 2 years are necessary to document the positive protective trend on BMD apparently associated with elinzanetant administration. Whether confirmatory, these preliminary studies would support definitive studies on real events, such as osteoporotic fractures.

## 8. Conclusions

Observational studies demonstrate that women experiencing frequent vasomotor symptoms or menopause-related complaints are at an increased risk for long-term health consequences. Women experiencing significant vasomotor or menopausal symptoms exhibit an increased prevalence of metabolic syndrome, cardiovascular events, and bone fractures. The observations suggest that these women exhibit an increased vulnerability to hormone withdrawal, resulting in more pronounced short- and long-term health effects. The present review hypothesizes that the presence of symptoms through biological modifications may contribute to increases in the risk of long-term health disease. It is hypothesized that the elevation of the heart rate, of nocturnal blood pressure, of 24 h cortisol production, and of oxidative stress documented in women with vasomotor and menopausal symptoms may represent the intermediate mechanisms contributing to the pathogenesis of various long-term health consequences impacting the cardiovascular system, bones, and even the brain. Limited interventional data indicating a reduction in 24 h urinary cortisol and blood pressure, in conjunction with an improvement in menopausal symptoms induced by non-hormonal therapies, seems to support this possibility. The selective neurokinin antagonists, fezolinetant and elinzanetant, alleviate symptoms and may potentially disrupt mechanisms that contribute to the burden of risk factors for long-term chronic conditions, including cardiovascular disease, osteoporosis, and cognitive impairment. Studies are needed to test this possibility, with relatively short-term clinical investigations possibly giving confirmatory results on this issue. Women who are either unwilling or have contraindication to the use of menopause hormone therapy may possibly find neurokinin antagonists not only useful for their short-term disturbances but also for the related long-term health consequences.

## Figures and Tables

**Table 1:** Summary of evidence linking menopausal symptoms to long-term health consequences.

Health Consequence	Associated Menopausal Symptom/Factor	Key Findings
**Cardiovascular Disease (CVD)**	Frequent Vasomotor Symptoms (VMSs)	- Increased BMI, cholesterol, and blood pressure. - Impaired endothelial function and arterial calcification. - Elevated risk of cardiovascular events and mortality. - Increased visceral adiposity, inflammation, oxidative stress, and insulin resistance.
	Sleep Disruption	- Increased metabolic syndrome. - Increased nocturnal blood pressure and hypertension. - Heightened risk of CVD.
	Depressive Symptoms	- Increased metabolic syndrome. - Heightened risk of CVD.
**Osteoporosis**	Frequent Vasomotor Symptoms (VMSs)	- Heightened bone turnover and bone loss during menopausal transition. - Lower bone mineral density post-menopause. - Higher incidence of bone fractures.
	Sleep Disruption	- Imbalance in bone metabolism, leading to decreased bone mineral density. - Higher risk of bone fractures.
	Depression	- Decreased bone mineral density. - Increased risk of osteoporosis.
**Cognitive Decline**	Vasomotor Symptoms (VMSs)	- Impaired short-term memory. - Nighttime VMSs are associated with brain white matter hyperintensities, a marker for neurodegenerative disease risk.
	Sleep Disruption	- Impaired short-term memory.

## Data Availability

Not applicable.

## Abbreviations

The following abbreviations are used in this manuscript:

ACTH	Adrenocorticotropic Hormone
BMI	Body Mass Index
BP	Blood Pressure
GABA	Gamma-Aminobutyric Acid
KNDy	Kisspeptin-Neurokinin-Dynorphin
LH	Luteinizing Hormone
SSRI	Selective Serotonin Reuptake Inhibitors
WHI	Women’s Health Initiative

## References

[1] Gast G.C.M., Grobbee D.E., Pop V.J.M., Keyzer J.J., Wijnands-van Gent C.J.M., Samsioe G.N., Nilsson P.M., van der Schouw Y.T. (2008). Menopausal Complaints Are Associated with Cardiovascular Risk Factors. Hypertension.

[2] Thurston R.C., Sutton-Tyrrell K., Everson-Rose S.A., Hess R., Matthews K.A. (2008). Hot Flashes and Subclinical Cardiovascular Disease: Findings from the Study of Women’s Health Across the Nation Heart Study. Circulation.

[3] Gallicchio L., Miller S.R., Zacur H., Flaws J.A. (2010). Hot Flashes and Blood Pressure in Midlife Women. Maturitas.

[4] Dam V., Dobson A.J., Onland-Moret N.C., van der Schouw Y.T., Mishra G.D. (2020). Vasomotor Menopausal Symptoms and Cardiovascular Disease Risk in Midlife: A Longitudinal Study. Maturitas.

[5] Hyvärinen M., Karvanen J., Juppi H.K., Karppinen J.E., Tammelin T.H., Kovanen V., Aukee P., Sipilä S., Rantalainen T., Laakkonen E.K. (2023). Menopausal Symptoms and Cardiometabolic Risk Factors in Middle-Aged Women: A Cross-Sectional and Longitudinal Study with 4-Year Follow-Up. Maturitas.

[6] Gast G.C.M., Samsioe G.N., Grobbee D.E., Nilsson P.M., van der Schouw Y.T. (2010). Vasomotor Symptoms, Estradiol Levels and Cardiovascular Risk Profile in Women. Maturitas.

[7] Sassarini J., Fox H., Ferrell W., Sattar N., Lumsden M.A. (2011). Vascular Function and Cardiovascular Risk Factors in Women with Severe Flushing. Clin. Endocrinol..

[8] Franco O.H., Muka T., Colpani V., Kunutsor S., Chowdhury S., Chowdhury R., Kavousi M. (2015). Vasomotor Symptoms in Women and Cardiovascular Risk Markers: Systematic Review and Meta-Analysis. Maturitas.

[9] Biglia N., Cagnacci A., Gambacciani M., Lello S., Maffei S., Nappi R.E. (2017). Vasomotor Symptoms in Menopause: A Biomarker of Cardiovascular Disease Risk and Other Chronic Diseases?. Climacteric.

[10] Svartberg J., von Mühlen D., Kritz-Silverstein D., Barrett-Connor E. (2009). Vasomotor Symptoms and Mortality: The Rancho Bernardo Study. Menopause.

[11] Szmuilowicz E.D., Manson J.E., Rossouw J.E., Howard B.V., Margolis K.L., Greep N.C., Brzyski R.G., Stefanick M.L., O’Sullivan M.J., Wu C. (2011). Vasomotor Symptoms and Cardiovascular Events in Postmenopausal Women. Menopause.

[12] Muka T., Oliver-Williams C., Colpani V., Kunutsor S., Chowdhury S., Chowdhury R., Kavousi M., Franco O.H. (2016). Association of Vasomotor and Other Menopausal Symptoms with Risk of Cardiovascular Disease: A Systematic Review and Meta-Analysis. PLoS ONE.

[13] Ferri L.A., Morici N., Bassanelli G., Franco N., Misuraca L., Lenatti L., Jacono E.L., Leuzzi C., Corrada E., Aranzulla T.C. (2018). History of Vasomotor Symptoms, Extent of Coronary Artery Disease, and Clinical Outcomes after Acute Coronary Syndrome in Postmenopausal Women. Menopause.

[14] Zhu D., Chung H.F., Dobson A.J., Pandeya N., Anderson D.J., Kuh D., Hardy R., Brunner E.J., Avis N.E., Gold E.B. (2020). Vasomotor Menopausal Symptoms and Risk of Cardiovascular Disease: A Pooled Analysis of Six Prospective Studies. Am. J. Obstet. Gynecol..

[15] Nudy M., Jiang X., Aragaki A.K., Manson J.E., Shadyab A.H., Foy A.J., Buerger J., Kelsey A.M., LeBlanc E.S., Wild R.A. (2020). The Severity of Vasomotor Symptoms and Number of Menopausal Symptoms in Postmenopausal Women and Select Clinical Health Outcomes in the Women’s Health Initiative Calcium and Vitamin D Randomized Clinical Trial. Menopause.

[16] Thurston R.C., Johnson B.D., Shufelt C.L., Braunstein G.D., Berga S.L., Stanczyk F.Z., Pepine C.J., Bittner V., Reis S.E., Thompson D.V. (2017). Menopausal Symptoms and Cardiovascular Disease Mortality in the Women’s Ischemia Syndrome Evaluation (WISE). Menopause.

[17] Thurston R.C., El Khoudary S.R., Sutton-Tyrrell K., Crandall C.J., Gold E., Sternfeld B., Selzer F., Matthews K.A. (2011). Are Vasomotor Symptoms Associated with Alterations in Hemostatic and Inflammatory Markers? Findings from the Study of Women’s Health Across the Nation. Menopause.

[18] Crandall C.J., Tseng C.H., Crawford S.L., Thurston R.C., Gold E.B., Johnston J.M., Greendale G.A. (2011). Association of Menopausal Vasomotor Symptoms with Increased Bone Turnover during the Menopausal Transition. J. Bone Miner. Res..

[19] Gast G.C.M., Grobbee D.E., Pop V.J.M., Keyzer J.J., Wijnands-van Gent C.J.M., Samsioe G.N., Nilsson P.M., van der Schouw Y.T. (2009). Vasomotor Symptoms Are Associated with a Lower Bone Mineral Density. Menopause.

[20] Crandall C.J., Zheng Y., Crawford S.L., Thurston R.C., Gold E.B., Johnston J.M., Greendale G.A. (2009). Presence of Vasomotor Symptoms Is Associated with Lower Bone Mineral Density: A Longitudinal Analysis. Menopause.

[21] Crandall C.J., Aragaki A., Cauley J.A., Manson J.E., LeBlanc E., Wallace R., Wactawski-Wende J., LaCroix A., O’Sullivan M.J., Vitolins M. (2015). Associations of Menopausal Vasomotor Symptoms with Fracture Incidence. J. Clin. Endocrinol. Metab..

[22] Maki P.M., Drogos L.L., Rubin L.H., Banuvar S., Shulman L.P., Geller S.E. (2008). Objective Hot Flashes Are Negatively Related to Verbal Memory Performance in Midlife Women. Menopause.

[23] Drogos L.L., Rubin L.H., Geller S.E., Banuvar S., Shulman L.P., Maki P.M. (2013). Objective Cognitive Performance Is Related to Subjective Memory Complaints in Midlife Women with Moderate to Severe Vasomotor Symptoms. Menopause.

[24] Thurston R.C., Aizenstein H.J., Derby C.A., Sejdić E., Maki P.M. (2016). Menopausal Hot Flashes and White Matter Hyperintensities. Menopause.

[25] Thurston R.C., Wu M., Aizenstein H.J., Chang Y., Barinas Mitchell E., Derby C.A., Maki P.M. (2020). Sleep Characteristics and White Matter Hyperintensities among Midlife Women. Sleep.

[26] Cagnacci A., Palma F., Napolitano A., Xholli A. (2017). Association between Pelvic Organ Prolapse and Climacteric Symptoms in Postmenopausal Women. Maturitas.

[27] Cagnacci A., Palma F., Carbone M.M., Grandi G., Xholli A. (2017). Association between Urinary Incontinence and Climacteric Symptoms in Postmenopausal Women. Menopause.

[28] van der Schouw Y.T., Grobbee D.E. (2005). Menopausal Complaints, Oestrogens, and Heart Disease Risk: An Explanation for Discrepant Findings on the Benefits of Post-Menopausal Hormone Therapy. Eur. Heart J..

[29] Sturdee D.W., Wilson K.A., Pipili E., Crocker A.D. (1978). Physiological Aspects of Menopausal Hot Flush. Br. Med. J..

[30] Tataryn I.V., Lomax P., Bajorek J.G., Chesarek W., Meldrum D.R., Judd H.L. (1980). Postmenopausal Hot Flushes: A Disorder of Thermoregulation. Maturitas.

[31] Freedman R.R. (2001). Physiology of Hot Flashes. Am. J. Human Biol..

[32] Gombert-Labedens M., Vesterdorf K., Fuller A., Maloney S.K., Baker F.C. (2025). Effects of Menopause on Temperature Regulation. Temperature.

[33] Gold E.B., Colvin A., Avis N., Bromberger J., Greendale G.A., Powell L., Sternfeld B., Matthews K. (2006). Longitudinal Analysis of the Association between Vasomotor Symptoms and Race/Ethnicity across the Menopausal Transition: Study of Women’s Health across the Nation. Am. J. Public Health.

[34] Mishra G.D., Kuh D. (2012). Health Symptoms during Midlife in Relation to Menopausal Transition: British Prospective Cohort Study. BMJ.

[35] Avis N.E., Crawford S.L., Greendale G., Bromberger J.T., Everson-Rose S.A., Gold E.B., Hess R., Joffe H., Kravitz H.M., Tepper P.G. (2015). Duration of Menopausal Vasomotor Symptoms over the Menopause Transition. JAMA Intern. Med..

[36] Thurston R.C., El Khoudary S.R., Tepper P.G., Jackson E.A., Joffe H., Chen H.Y., Matthews K.A. (2016). Trajectories of Vasomotor Symptoms and Carotid Intima Media Thickness in the Study of Women’s Health Across the Nation. Stroke.

[37] Kravitz H.M., Ganz P.A., Bromberger J., Powell L.H., Sutton-Tyrrell K., Meyer P.M. (2003). Sleep Difficulty in Women at Midlife: A Community Survey of Sleep and the Menopausal Transition. Menopause.

[38] Kravitz H.M., Joffe H. (2011). Sleep during the Perimenopause: A SWAN Story. Obstet. Gynecol. Clin. N. Am..

[39] Xu H., Thurston R.C., Matthews K.A., Bryce C.L., Hays R.D., Kapoor W.N., Ness R.B., Hess R. (2012). Are Hot Flashes Associated with Sleep Disturbance during Midlife? Results from the STRIDE Cohort Study. Maturitas.

[40] Beverly Hery C.M., Hale L., Naughton M.J. (2020). Contributions of the Women’s Health Initiative to Understanding Associations between Sleep Duration, Insomnia Symptoms, and Sleep-Disordered Breathing across a Range of Health Outcomes in Postmenopausal Women. Sleep Health.

[41] Erlik Y., Tataryn I.V., Meldrum D.R., Lomax P., Bajorek J.G., Judd H.L. (1981). Association of Waking Episodes with Menopausal Hot Flushes. JAMA.

[42] Szymusiak R., Gvilia I., McGinty D. (2007). Hypothalamic Control of Sleep. Sleep Med..

[43] Rance N.E., Dacks P.A., Mittelman-Smith M.A., Romanovsky A.A., Krajewski-Hall S.J. (2013). Modulation of Body Temperature and LH Secretion by Hypothalamic KNDy (Kisspeptin, Neurokinin B and Dynorphin) Neurons: A Novel Hypothesis on the Mechanism of Hot Flushes. Front. Neuroendocr..

[44] Cagnacci A., Soldani R., Yen S.S. (1997). Melatonin Enhances Cortisol Levels in Aged Women: Reversible by Estrogens. J. Pineal Res..

[45] Bromberger J.T., Matthews K.A., Schott L.L., Brockwell S., Avis N.E., Kravitz H.M., Everson-Rose S.A., Gold E.B., Sowers M., Randolph J.F. (2007). Depressive Symptoms during the Menopausal Transition: The Study of Women’s Health Across the Nation (SWAN). J. Affect. Disord..

[46] Worsley R., Bell R.J., Gartoulla P., Robinson P.J., Davis S.R. (2017). Moderate-Severe Vasomotor Symptoms Are Associated with Moderate-Severe Depressive Symptoms. J. Womens Health.

[47] Vaccaro C.M., Capozzi A., Ettore G., Bernorio R., Cagnacci A., Gambacciani M., Coletta V., Maffei S., Nappi R.E., Scambia G. (2021). What Women Think about Menopause: An Italian Survey. Maturitas.

[48] Fidecicchi T., Giannini A., Chedraui P., Luisi S., Battipaglia C., Genazzani A.R., Genazzani A.D., Simoncini T. (2024). Neuroendocrine Mechanisms of Mood Disorders during Menopause Transition: A Narrative Review and Future Perspectives. Maturitas.

[49] Natari R.B., Clavarino A.M., McGuire T.M., Dingle K.D., Hollingworth S.A. (2018). The Bidirectional Relationship between Vasomotor Symptoms and Depression across the Menopausal Transition: A Systematic Review of Longitudinal Studies. Menopause.

[50] Gibson C.J., Ajmera M., O’Sullivan F., Shiozawa A., Lozano-Ortega G., Badillo E.C., Venkataraman M., Mancuso S. (2025). A Systematic Review of Anxiety and Depressive Symptoms Among Women Experiencing Vasomotor Symptoms Across Reproductive Stages in the US. Int. J. Womens Health.

[51] Riemann D., Workshop Participants (2009). Does Effective Management of Sleep Disorders Reduce Depressive Symptoms and the Risk of Depression?. Drugs.

[52] Baglioni C., Battagliese G., Feige B., Spiegelhalder K., Nissen C., Voderholzer U., Lombardo C., Riemann D. (2011). Insomnia as a Predictor of Depression: A Meta-Analytic Evaluation of Longitudinal Epidemiological Studies. J. Affect. Disord..

[53] Zeng Y., Liu T., Qiu R., Lian Q. (2025). Association among Objective and Subjective Sleep Duration, Depressive Symptoms and All-Cause Mortality: The Pathways Study. BMC Psychiatry.

[54] Cagnacci A., Xholli A., Sclauzero M., Venier M., Palma F., Gambacciani M., Writing Group of the ANGEL Study (2019). Vaginal Atrophy across the Menopausal Age: Results from the ANGEL Study. Climacteric.

[55] Cagnacci A., Venier M., Xholli A., Paglietti C., Caruso S., Angel Study (2020). Female Sexuality and Vaginal Health across the Menopausal Age. Menopause.

[56] Cagnacci A., Sclauzero M., Meriggiola C., Xholli A., ANGEL Study (2020). Lower Urinary Tract Symptoms and Their Relation to Vaginal Atrophy in Women across the Menopausal Age Span. Results from the ANGEL Multicentre Observational Study. Maturitas.

[57] Løkkegaard E., Jovanovic Z., Heitmann B.L., Keiding N., Ottesen B., Pedersen A.T. (2006). The Association between Early Menopause and Risk of Ischaemic Heart Disease: Influence of Hormone Therapy. Maturitas.

[58] Rivera C.M., Grossardt B.R., Rhodes D.J., Brown R.D., Roger V.L., Melton L.J., Rocca W.A. (2009). Increased Cardiovascular Mortality after Early Bilateral Oophorectomy. Menopause.

[59] Parker W.H., Broder M.S., Chang E., Feskanich D., Farquhar C., Liu Z., Shoupe D., Berek J.S., Hankinson S., Manson J.E. (2009). Ovarian Conservation at the Time of Hysterectomy and Long-Term Health Outcomes in the Nurses’ Health Study. Obstet. Gynecol..

[60] Maclaran K., Horner E., Panay N. (2010). Premature Ovarian Failure: Long-Term Sequelae. Menopause Int..

[61] Panay N., Anderson R.A., Nappi R.E., Vincent A.J., Vujovic S., Webber L., Wolfman W. (2020). Premature Ovarian Insufficiency: An International Menopause Society White Paper. Climacteric.

[62] Liu J., Jin X., Chen W., Wang L., Feng Z., Huang J. (2023). Early Menopause Is Associated with Increased Risk of Heart Failure and Atrial Fibrillation: A Systematic Review and Meta-Analysis. Maturitas.

[63] van der Schouw Y.T., van der Graaf Y., Steyerberg E.W., Eijkemans J.C., Banga J.D. (1996). Age at Menopause as a Risk Factor for Cardiovascular Mortality. Lancet.

[64] Janssen I., Powell L.H., Crawford S., Lasley B., Sutton-Tyrrell K. (2008). Menopause and the Metabolic Syndrome: The Study of Women’s Health Across the Nation. Arch. Intern. Med..

[65] Reeves A.N., Elliott M.R., Brooks M.M., Karvonen-Gutierrez C.A., Bondarenko I., Hood M.M., Harlow S.D. (2021). Symptom Clusters Predict Risk of Metabolic-Syndrome and Diabetes in Midlife: The Study of Women’s Health Across the Nation. Ann. Epidemiol..

[66] Ou Y.J., Lee J.I., Huang S.P., Chen S.C., Geng J.H., Su C.H. (2023). Association between Menopause, Postmenopausal Hormone Therapy and Metabolic Syndrome. J. Clin. Med..

[67] Thurston R.C., Sowers M.R., Sternfeld B., Gold E.B., Bromberger J., Chang Y., Joffe H., Crandall C.J., Waetjen L.E., Matthews K.A. (2009). Gains in Body Fat and Vasomotor Symptom Reporting over the Menopausal Transition: The Study of Women’s Health across the Nation. Am. J. Epidemiol..

[68] Doshi S.B., Agarwal A. (2013). The Role of Oxidative Stress in Menopause. J. Midlife Health.

[69] Gordon J.L., Rubinow D.R., Thurston R.C., Paulson J., Schmidt P.J., Girdler S.S. (2016). Cardiovascular, Hemodynamic, Neuroendocrine, and Inflammatory Markers in Women with and without Vasomotor Symptoms. Menopause.

[70] Sánchez-Rodríguez M.A., Zacarías-Flores M., Arronte-Rosales A., Mendoza-Núñez V.M. (2019). Association between Hot Flashes Severity and Oxidative Stress among Mexican Postmenopausal Women: A Cross-Sectional Study. PLoS ONE.

[71] Sadeghi M., Khalili M., Pourmoghaddas M., Talaei M. (2012). The Correlation between Blood Pressure and Hot Flashes in Menopausal Women. ARYA Atheroscler..

[72] Jackson E.A., El Khoudary S.R., Crawford S.L., Matthews K., Joffe H., Chae C., Thurston R.C. (2016). Hot Flash Frequency and Blood Pressure: Data from the Study of Women’s Health Across the Nation. J. Womens Health.

[73] Thurston R.C., El Khoudary S.R., Sutton-Tyrrell K., Crandall C.J., Sternfeld B., Joffe H., Gold E.B., Selzer F., Matthews K.A. (2012). Vasomotor Symptoms and Insulin Resistance in the Study of Women’s Health across the Nation. J. Clin. Endocrinol. Metab..

[74] Gray K.E., Katon J.G., LeBlanc E.S., Woods N.F., Bastian L.A., Reiber G.E., Weitlauf J.C., Nelson K.M., LaCroix A.Z. (2018). Vasomotor Symptom Characteristics: Are They Risk Factors for Incident Diabetes?. Menopause.

[75] Bechlioulis A., Kalantaridou S.N., Naka K.K., Chatzikyriakidou A., Calis K.A., Makrigiannakis A., Papanikolaou O., Kaponis A., Katsouras C., Georgiou I. (2010). Endothelial Function, but Not Carotid Intima-Media Thickness, Is Affected Early in Menopause and Is Associated with Severity of Hot Flushes. J. Clin. Endocrinol. Metab..

[76] Thurston R.C., Chang Y., Barinas-Mitchell E., Jennings J.R., Landsittel D.P., Santoro N., von Känel R., Matthews K.A. (2016). Menopausal Hot Flashes and Carotid Intima Media Thickness Among Midlife Women. Stroke.

[77] Thurston R.C., Kuller L.H., Edmundowicz D., Matthews K.A. (2018). History of Hot Flashes and Aortic Calcification among Postmenopausal Women. Menopause.

[78] Nilsson S., Qvick A., Henriksson M., Lawesson S.S., Holm A.C.S., Leander K. (2024). Menopausal Vasomotor Symptoms and Subclinical Atherosclerotic Cardiovascular Disease: A Population-Based Study. J. Am. Heart Assoc..

[79] Thurston R.C., Aslanidou Vlachos H.E., Derby C.A., Jackson E.A., Brooks M.M., Matthews K.A., Harlow S., Joffe H., El Khoudary S.R. (2021). Menopausal Vasomotor Symptoms and Risk of Incident Cardiovascular Disease Events in SWAN. J. Am. Heart Assoc..

[80] Chasens E.R., Imes C.C., Kariuki J.K., Luyster F.S., Morris J.L., DiNardo M.M., Godzik C.M., Jeon B., Yang K. (2021). Sleep and Metabolic Syndrome. Nurs. Clin. N. Am..

[81] Haghayegh S., Strohmaier S., Hamaya R., Eliassen A.H., Willett W.C., Rimm E.B., Schernhammer E.S. (2023). Sleeping Difficulties, Sleep Duration, and Risk of Hypertension in Women. Hypertension.

[82] Tomitani N., Hoshide S., Kario K. (2024). Sleep and Hypertension-up to Date 2024. Hypertens. Res..

[83] Thurston R.C., Chang Y., von Känel R., Barinas-Mitchell E., Jennings J.R., Hall M.H., Santoro N., Buysse D.J., Matthews K.A. (2017). Sleep Characteristics and Carotid Atherosclerosis Among Midlife Women. Sleep.

[84] Medic G., Wille M., Hemels M.E. (2017). Short- and Long-Term Health Consequences of Sleep Disruption. Nat. Sci. Sleep.

[85] Zhang M., Chen J., Yin Z., Wang L., Peng L. (2021). The Association between Depression and Metabolic Syndrome and Its Components: A Bidirectional Two-Sample Mendelian Randomization Study. Transl. Psychiatry.

[86] Prigge R., Wild S.H., Jackson C.A. (2022). Depression, Diabetes, Comorbid Depression and Diabetes and Risk of All-Cause and Cause-Specific Mortality: A Prospective Cohort Study. Diabetologia.

[87] Cagnacci A., Cannoletta M., Caretto S., Zanin R., Xholli A., Volpe A. (2011). Increased Cortisol Level: A Possible Link between Climacteric Symptoms and Cardiovascular Risk Factors. Menopause.

[88] Cagnacci A., Cannoletta M., Palma F., Zanin R., Xholli A., Volpe A. (2012). Menopausal Symptoms and Risk Factors for Cardiovascular Disease in Postmenopause. Climacteric.

[89] Cagnacci A., Gambera A., Bonaccorsi G., Xholli A., ANGEL Study (2022). Relation between Blood Pressure and Genito-Urinary Symptoms in the Years across the Menopausal Age. Climacteric.

[90] Cagnacci A., Palma F., Romani C., Xholli A., Bellafronte M., Di Carlo C. (2015). Are Climacteric Complaints Associated with Risk Factors of Cardiovascular Disease in Peri-Menopausal Women?. Gynecol. Endocrinol..

[91] Cagnacci A., Cannoletta M., Palma F., Bellafronte M., Romani C., Palmieri B. (2015). Relation between Oxidative Stress and Climacteric Symptoms in Early Postmenopausal Women. Climacteric.

[92] Nudy M., Aragaki A.K., Jiang X., Manson J.E., Allison M.A., Shadyab A.H., Hodis H.N., Wild R.A., Robbins J.A., Liu S. (2022). The Severity of Individual Menopausal Symptoms, Cardiovascular Disease, and All-Cause Mortality in the Women’s Health Initiative Observational Cohort. Menopause.

[93] Pansini F., Bagni B., Bonaccorsi G., Albertazzi P., Zanotti L., Farina A., Campobasso C., Orlandi R., Mollica G. (1995). Oophorectomy and Spine Bone Density: Evidence of a Higher Rate of Bone Loss in Surgical Compared with Spontaneous Menopause. Menopause.

[94] Pouillès J.M., Trémollières F., Bonneu M., Ribot C. (1994). Influence of Early Age at Menopause on Vertebral Bone Mass. J. Bone Miner. Res..

[95] Ohta H., Sugimoto I., Masuda A., Komukai S., Suda Y., Makita K., Takamatsu K., Horiguchi F., Nozawa S. (1996). Decreased Bone Mineral Density Associated with Early Menopause Progresses for at Least Ten Years: Cross-Sectional Comparisons between Early and Normal Menopausal Women. Bone.

[96] Kotsopoulos J., Hall E., Finch A., Hu H., Murphy J., Rosen B., Narod S.A., Cheung A.M. (2019). Changes in Bone Mineral Density After Prophylactic Bilateral Salpingo-Oophorectomy in Carriers of a BRCA Mutation. JAMA Netw. Open.

[97] van Der Voort D.J.M., van Der Weijer P.H.M., Barentsen R. (2003). Early Menopause: Increased Fracture Risk at Older Age. Osteoporos. Int..

[98] Finkelstein J.S., Brockwell S.E., Mehta V., Greendale G.A., Sowers M.R., Ettinger B., Lo J.C., Johnston J.M., Cauley J.A., Danielson M.E. (2008). Bone Mineral Density Changes during the Menopause Transition in a Multiethnic Cohort of Women. J. Clin. Endocrinol. Metab..

[99] Swanson C.M. (2021). Sleep Disruptions and Bone Health: What Do We Know so Far?. Curr. Opin. Endocrinol. Diabetes Obes..

[100] Swanson C.M., Shea S.A., Kohrt W.M., Wright K.P., Cain S.W., Munch M., Vujović N., Czeisler C.A., Orwoll E.S., Buxton O.M. (2020). Sleep Restriction with Circadian Disruption Negatively Alter Bone Turnover Markers in Women. J. Clin. Endocrinol. Metab..

[101] Cherian K.E., Kapoor N., Paul T.V. (2022). Disrupted Sleep Architecture Is Associated with Incident Bone Loss in Indian Postmenopausal Women: A Prospective Study. J. Bone Miner. Res..

[102] Cauley J.A., Kravitz H.M., Ruppert K., Lian Y., Hall M.J., Harlow S.D., Finkelstein J.S., Greendale G. (2023). Self-Reported Sleep Disturbances over the Menopausal Transition and Fracture Risk: The Study of Women’s Health Across the Nation. JBMR Plus.

[103] Altindag O., Altindag A., Asoglu M., Gunes M., Soran N., Deveci Z. (2007). Relation of Cortisol Levels and Bone Mineral Density among Premenopausal Women with Major Depression. Int. J. Clin. Pract..

[104] Eskandari F., Martinez P.E., Torvik S., Phillips T.M., Sternberg E.M., Mistry S., Ronsaville D., Wesley R., Toomey C., Sebring N.G. (2007). Low Bone Mass in Premenopausal Women with Depression. Arch. Intern. Med..

[105] Petronijević M., Petronijević N., Ivković M., Stefanović D., Radonjić N., Glisić B., Ristić G., Damjanović A., Paunović V. (2008). Low Bone Mineral Density and High Bone Metabolism Turnover in Premenopausal Women with Unipolar Depression. Bone.

[106] Aydin H., Mutlu N., Akbas N.B.G. (2011). Treatment of a Major Depression Episode Suppresses Markers of Bone Turnover in Premenopausal Women. J. Psychiatr. Res..

[107] Atteritano M., Lasco A., Mazzaferro S., Macrì I., Catalano A., Santangelo A., Bagnato G., Bagnato G., Frisina N. (2013). Bone Mineral Density, Quantitative Ultrasound Parameters and Bone Metabolism in Postmenopausal Women with Depression. Intern. Emerg. Med..

[108] Thurston R.C., Christie I.C., Matthews K.A. (2010). Hot Flashes and Cardiac Vagal Control: A Link to Cardiovascular Risk?. Menopause.

[109] Gupta P., Harte A.L., da Silva N.F., Khan H., Barnett A.H., Kumar S., Sturdee D.W., McTernan P.G. (2007). Expression of Calcitonin Gene-Related Peptide, Adrenomedullin, and Receptor Modifying Proteins in Human Adipose Tissue and Alteration in Their Expression with Menopause Status. Menopause.

[110] Custodis F., Baumhäkel M., Schlimmer N., List F., Gensch C., Böhm M., Laufs U. (2008). Heart Rate Reduction by Ivabradine Reduces Oxidative Stress, Improves Endothelial Function, and Prevents Atherosclerosis in Apolipoprotein E-deficient Mice. Circulation.

[111] Whelton S.P., Blankstein R., Al-Mallah M.H., Lima J.A.C., Bluemke D.A., Hundley W.G., Polak J.F., Blumenthal R.S., Nasir K., Blaha M.J. (2013). Association of Resting Heart Rate with Carotid and Aortic Arterial Stiffness: Multi-Ethnic Study of Atherosclerosis. Hypertension.

[112] Caetano J., Delgado Alves J. (2015). Heart Rate and Cardiovascular Protection. Eur. J. Intern. Med..

[113] Seviiri M., Lynch B.M., Hodge A.M., Yang Y., Liew D., English D.R., Giles G.G., Milne R.L., Dugué P.A. (2018). Resting Heart Rate, Temporal Changes in Resting Heart Rate, and Overall and Cause-Specific Mortality. Heart.

[114] de la Sierra A., Staplin N., Ruilope L.M., Gorostidi M., Vinyoles E., Segura J., Baigent C., Williams B. (2024). A Blunted Nocturnal Blood Pressure Decline Is Associated with All-Cause and Cardiovascular Mortality. J. Hypertens..

[115] Lempiäinen P.A., Ylitalo A., Huikuri H., Kesäniemi Y.A., Ukkola O.H. (2024). Non-Dipping Blood Pressure Pattern Is Associated with Cardiovascular Events in a 21-Year Follow-up Study. J. Human Hypertens..

[116] Parati G., Pengo M.F., Avolio A., Azizi M., Bothe T.L., Burnier M., Cappuccio F.P., Sierra A.D.L., Fava C., Gironacci M.M. (2025). Nocturnal Blood Pressure: Pathophysiology, Measurement and Clinical Implications. Position Paper of the European Society of Hypertension. J. Hypertens..

[117] Woods N.F., Carr M.C., Tao E.Y., Taylor H.J., Mitchell E.S. (2006). Increased Urinary Cortisol Levels during the Menopausal Transition. Menopause.

[118] Cagnacci A., Soldani R., Yen S.S. (1995). Melatonin Enhances Cortisol Levels in Aged but Not Young Women. Eur. J. Endocrinol..

[119] Casper R.F., Yen S.S., Wilkes M.M. (1979). Menopausal Flushes: A Neuroendocrine Link with Pulsatile Luteninizing Hormone Secreation. Science.

[120] Meldrum D.R., Tataryn I.V., Frumar A.M., Erlik Y., Lu K.H., Judd H.L. (1980). Gonadotropins, Estrogens, and Adrenal Steroids during the Menopausal Hot Flash. J. Clin. Endocrinol. Metab..

[121] Genazzani A.R., Petraglia F., Facchinetti F., Facchini V., Volpe A., Alessandrini G. (1984). Increase of Proopiomelanocortin-Related Peptides during Subjective Menopausal Flushes. Am. J. Obstet. Gynecol..

[122] Gibson C.J., Thurston R.C., Matthews K.A. (2016). Cortisol Dysregulation Is Associated with Daily Diary-Reported Hot Flashes among Midlife Women. Clin. Endocrinol..

[123] Reed S.D., Newton K.M., Larson J.C., Booth-LaForce C., Woods N.F., Landis C.A., Tolentino E., Carpenter J.S., Freeman E.W., Joffe H. (2016). Daily Salivary Cortisol Patterns in Midlife Women with Hot Flashes. Clin. Endocrinol..

[124] Morgan E., Schumm L.P., McClintock M., Waite L., Lauderdale D.S. (2017). Sleep Characteristics and Daytime Cortisol Levels in Older Adults. Sleep.

[125] Pulopulos M.M., Hidalgo V., Puig-Perez S., Montoliu T., Salvador A. (2020). Relationship between Cortisol Changes during the Night and Subjective and Objective Sleep Quality in Healthy Older People. Int. J. Environ. Res. Public Health.

[126] Chen Y., Xu W., Chen Y., Gong J., Wu Y., Chen S., He Y., Yu H., Xie L. (2024). The Effect of Acute Sleep Deprivation on Cortisol Level: A Systematic Review and Meta-Analysis. Endocr. J..

[127] Sahola N., Toffol E., Kalleinen N., Polo-Kantola P. (2024). Worse Sleep Architecture but Not Self-Reported Insomnia and Sleepiness Is Associated with Higher Cortisol Levels in Menopausal Women. Maturitas.

[128] Mortola J.F., Liu J.H., Gillin J.C., Rasmussen D.D., Yen S.S. (1987). Pulsatile Rhythms of Adrenocorticotropin (ACTH) and Cortisol in Women with Endogenous Depression: Evidence for Increased ACTH Pulse Frequency. J. Clin. Endocrinol. Metab..

[129] Bertollo A.G., Santos C.F., Bagatini M.D., Ignácio Z.M. (2025). Hypothalamus-Pituitary-Adrenal and Gut-Brain Axes in Biological Interaction Pathway of the Depression. Front. Neurosci..

[130] Pasquali R., Vicennati V., Cacciari M., Pagotto U. (2006). The Hypothalamic-Pituitary-Adrenal Axis Activity in Obesity and the Metabolic Syndrome. Ann. N. Y. Acad. Sci..

[131] Esteghamati A., Morteza A., Khalilzadeh O., Noshad S., Novin L., Nakhjavani M. (2011). Association of Serum Cortisol Levels with Parameters of Metabolic Syndrome in Men and Women. Clin. Investig. Med..

[132] Martocchia A., Stefanelli M., Falaschi G.M., Toussan L., Ferri C., Falaschi P. (2016). Recent Advances in the Role of Cortisol and Metabolic Syndrome in Age-Related Degenerative Diseases. Aging Clin. Exp. Res..

[133] Mazgelytė E., Karčiauskaitė D. (2024). Cortisol in Metabolic Syndrome. Adv. Clin. Chem..

[134] Jutla S.K., Yuyun M.F., Quinn P.A., Ng L.L. (2014). Plasma Cortisol and Prognosis of Patients with Acute Myocardial Infarction. J. Cardiovasc. Med..

[135] Jokinen J., Nordström P. (2009). HPA Axis Hyperactivity and Cardiovascular Mortality in Mood Disorder Inpatients. J. Affect. Disord..

[136] Vogelzangs N., Beekman A.T.F., Milaneschi Y., Bandinelli S., Ferrucci L., Penninx B.W.J.H. (2010). Urinary Cortisol and Six-Year Risk of All-Cause and Cardiovascular Mortality. J. Clin. Endocrinol. Metab..

[137] Kumari M., Shipley M., Stafford M., Kivimaki M. (2011). Association of Diurnal Patterns in Salivary Cortisol with All-Cause and Cardiovascular Mortality: Findings from the Whitehall II Study. J. Clin. Endocrinol. Metab..

[138] Reynolds R.M., Dennison E.M., Walker B.R., Syddall H.E., Wood P.J., Andrew R., Phillips D.I., Cooper C. (2005). Cortisol Secretion and Rate of Bone Loss in a Population-Based Cohort of Elderly Men and Women. Calcif. Tissue Int..

[139] Tauchmanovà L., Pivonello R., Di Somma C., Rossi R., De Martino M.C., Camera L., Klain M., Salvatore M., Lombardi G., Colao A. (2006). Bone Demineralization and Vertebral Fractures in Endogenous Cortisol Excess: Role of Disease Etiology and Gonadal Status. J. Clin. Endocrinol. Metab..

[140] Gonzalez Rodriguez E., Lamy O., Stoll D., Metzger M., Preisig M., Kuehner C., Vollenweider P., Marques-Vidal P., Waeber G., Aubry-Rozier B. (2017). High Evening Cortisol Level Is Associated with Low TBS and Increased Prevalent Vertebral Fractures: OsteoLaus Study. J. Clin. Endocrinol. Metab..

[141] Greendale G.A., Unger J.B., Rowe J.W., Seeman T.E. (1999). The Relation between Cortisol Excretion and Fractures in Healthy Older People: Results from the MacArthur Studies-Mac. J. Am. Geriatr. Soc..

[142] Yap Y., Tung N.Y.C., Shen L., Bei B., Phillips A., Wiley J.F. (2024). Daily Associations between Salivary Cortisol and Electroencephalographic-Assessed Sleep: A 15-Day Intensive Longitudinal Study. Sleep.

[143] Born J., Wagner U. (2004). Memory Consolidation during Sleep: Role of Cortisol Feedback. Ann. N. Y. Acad. Sci..

[144] Newcomer J.W., Selke G., Melson A.K., Hershey T., Craft S., Richards K., Alderson A.L. (1999). Decreased Memory Performance in Healthy Humans Induced by Stress-Level Cortisol Treatment. Arch. Gen. Psychiatry.

[145] Basta M., Vgontzas A.N., Fernandez-Mendoza J., Antypa D., Li Y., Zaganas I., Panagiotakis S., Karagkouni E., Simos P. (2022). Basal Cortisol Levels Are Increased in Patients with Mild Cognitive Impairment: Role of Insomnia and Short Sleep Duration. J. Alzheimers Dis..

[146] Hachul de Campos H., Brandão L.C., D’Almeida V., Grego B.H.C., Bittencourt L.R., Tufik S., Baracat E.C. (2006). Sleep Disturbances, Oxidative Stress and Cardiovascular Risk Parameters in Postmenopausal Women Complaining of Insomnia. Climacteric.

[147] Kolesnikova L.I., Semenova N.V., Solodova E.I., Madaeva I.M. (2017). Oxidative stress in women with insomnia in different stages of menopause. Ter. Arkhiv.

[148] Black C.N., Bot M., Scheffer P.G., Cuijpers P., Penninx B.W.J.H. (2015). Is Depression Associated with Increased Oxidative Stress? A Systematic Review and Meta-Analysis. Psychoneuroendocrinology.

[149] Liang G., Kow A.S.F., Yusof R., Tham C.L., Ho Y.C., Lee M.T. (2024). Menopause-Associated Depression: Impact of Oxidative Stress and Neuroinflammation on the Central Nervous System-A Review. Biomedicines.

[150] Yu Y., Yu T., Liu K., Li Y., Luan Y., Yang T., Li W., Cong H., Wu X. (2025). Perimenopausal Depression: Targeting Inflammation and Oxidative Stress (Review). Mol. Med. Rep..

[151] Nishiyama Y., Katsumata T., Otori T., Katayama Y. (2010). Carotid Hemodynamic Parameters Are Useful for Discriminating between Atherothrombotic Infarction and Lacunar Infarction. J. Stroke Cerebrovasc. Dis..

[152] Das U.N. (2003). Folic Acid Says NO to Vascular Diseases. Nutrition.

[153] Vassalle C., Petrozzi L., Botto N., Andreassi M.G., Zucchelli G.C. (2004). Oxidative Stress and Its Association with Coronary Artery Disease and Different Atherogenic Risk Factors. J. Intern. Med..

[154] Xiang D., Liu Y., Zhou S., Zhou E., Wang Y. (2021). Protective Effects of Estrogen on Cardiovascular Disease Mediated by Oxidative Stress. Oxid. Med. Cell. Longev..

[155] Yan Q., Liu S., Sun Y., Chen C., Yang S., Lin M., Long J., Yao J., Lin Y., Yi F. (2023). Targeting Oxidative Stress as a Preventive and Therapeutic Approach for Cardiovascular Disease. J. Transl. Med..

[156] Jin S., Wang H., Zhang X., Song M., Liu B., Sun W. (2024). Emerging Regulatory Mechanisms in Cardiovascular Disease: Ferroptosis. Biomed. Pharmacother..

[157] Cervellati C., Bonaccorsi G., Cremonini E., Romani A., Fila E., Castaldini M.C., Ferrazzini S., Giganti M., Massari L. (2014). Oxidative Stress and Bone Resorption Interplay as a Possible Trigger for Postmenopausal Osteoporosis. BioMed Res. Int..

[158] Shahriarpour Z., Nasrabadi B., Hejri-Zarifi S., Shariati-Bafghi S.E., Yousefian-Sanny M., Karamati M., Rashidkhani B. (2021). Oxidative Balance Score and Risk of Osteoporosis among Postmenopausal Iranian Women. Arch. Osteoporos..

[159] Malekian S., Mirghafourvand M., Najafipour F., Ostadrahimi A., Ghassab-Abdollahi N., Farshbaf-Khalili A. (2023). The Associations between Bone Mineral Density and Oxidative Stress Biomarkers in Postmenopausal Women. Korean J. Fam. Med..

[160] Yang S., Feskanich D., Willett W.C., Eliassen A.H., Wu T. (2014). Association between Global Biomarkers of Oxidative Stress and Hip Fracture in Postmenopausal Women: A Prospective Study. J. Bone Miner. Res..

[161] Baber R.J., Panay N., Fenton A., IMS Writing Group (2016). 2016 IMS Recommendations on Women’s Midlife Health and Menopause Hormone Therapy. Climacteric.

[162] Lambrinoudaki I., Armeni E., Goulis D., Bretz S., Ceausu I., Durmusoglu F., Erkkola R., Fistonic I., Gambacciani M., Geukes M. (2022). Menopause, Wellbeing and Health: A Care Pathway from the European Menopause and Andropause Society. Maturitas.

[163] Faubion S.S.M.C., Crandall C.J.M., Davis L.D., El Khoudary S.R.P., Hodis H.N., Lobo R.A., Maki P.M., Manson J.E.M., Pinkerton J.V.M., Santoro N.F. (2022). The 2022 Hormone Therapy Position Statement of The North American Menopause Society. Menopause.

[164] Palacios S., Rebelo C., Casquilho A., Costa A.R., Cagnacci A., Cano A., Castelo-Branco C., Di Carlo C., Romão F., Geraldes F. (2024). POESIT Recommendations on Management of Body-Identical Hormones in Menopausal Symptoms. Climacteric.

[165] Rossouw J.E., Prentice R.L., Manson J.E., Wu L., Barad D., Barnabei V.M., Ko M., LaCroix A.Z., Margolis K.L., Stefanick M.L. (2007). Postmenopausal Hormone Therapy and Risk of Cardiovascular Disease by Age and Years Since Menopause. JAMA.

[166] Cagnacci A., Venier M. (2019). The Controversial History of Hormone Replacement Therapy. Medicina.

[167] Rossouw J.E., Aragaki A.K., Manson J.E., Szmuilowicz E.D., Harrington L.B., Johnson K.C., Allison M., Haring B., Saquib N., Shadyab A.H. (2025). Menopausal Hormone Therapy and Cardiovascular Diseases in Women with Vasomotor Symptoms: A Secondary Analysis of the Women’s Health Initiative Randomized Clinical Trials. JAMA Intern. Med..

[168] Samsioe G. (1993). Hormone Replacement Therapy and Cardiovascular Disease. Int. J. Fertil. Menopausal Stud..

[169] Schierbeck L.L., Rejnmark L., Tofteng C.L., Stilgren L., Eiken P., Mosekilde L., Køber L., Jensen J.E.B. (2012). Effect of Hormon eReplacement Therapy on Cardiovascular Events in Recently Postmenopausal Women: Randomised Trial. BMJ.

[170] Gambacciani M., Spinetti A., Taponeco F., Cappagli B., Piaggesi L., Fioretti P. (1994). Longitudinal Evaluation of Perimenopausal Vertebral Bone Loss: Effects of a Low-Dose Oral Contraceptive Preparation on Bone Mineral Density and Metabolism. Obstet. Gynecol..

[171] Gambacciani M., Ciaponi M., Cappagli B., Benussi C., Genazzani A.R. (2000). Longitudinal Evaluation of Perimenopausal Femoral Bone Loss: Effects of a Low-Dose Oral Contraceptive Preparation on Bone Mineral Density and Metabolism. Osteoporos. Int..

[172] Cagnacci A., Melis G.B., Soldani R., Paoletti A.M., Gambacciani M., Spinetti A., Fioretti P. (1991). Neuroendocrine and Clinical Effects of Transdermal 17 Beta-Estradiol in Postmenopausal Women. Maturitas.

[173] Cauley J.A., Robbins J., Chen Z., Cummings S.R., Jackson R.D., LaCroix A.Z., LeBoff M., Lewis C.E., McGowan J., Neuner J. (2003). Effects of Estrogen plus Progestin on Risk of Fracture and Bone Mineral Density: The Women’s Health Initiative Randomized Trial. JAMA.

[174] Gambacciani M., Levancini M. (2014). Hormone Replacement Therapy and the Prevention of Postmenopausal Osteoporosis. Prz. Menopauzalny.

[175] Komesaroff P.A., Esler M.D., Sudhir K. (1999). Estrogen Supplementation Attenuates Glucocorticoid and Catecholamine Responses to Mental Stress in Perimenopausal Women. J. Clin. Endocrinol. Metab..

[176] Herrera A.Y., Hodis H.N., Mack W.J., Mather M. (2017). Estradiol Therapy After Menopause Mitigates Effects of Stress on Cortisol and Working Memory. J. Clin. Endocrinol. Metab..

[177] Cagnacci A., Rovati L., Zanni A., Malmusi S., Facchinetti F., Volpe A. (1999). Physiological Doses of Estradiol Decrease Nocturnal Blood Pressure in Normotensive Postmenopausal Women. Am. J. Physiol..

[178] Cannoletta M., Cagnacci A. (2014). Modification of Blood Pressure in Postmenopausal Women: Role of Hormone Replacement Therapy. Int. J. Womens Health.

[179] Cagnacci A., Xholli A., Fontanesi F., Neri I., Facchinetti F., Palma F. (2021). Treatment of Menopausal Symptoms: Concomitant Modification of Cortisol. Menopause.

[180] Befus D., Coeytaux R.R., Goldstein K.M., McDuffie J.R., Shepherd-Banigan M., Goode A.P., Kosinski A., Van Noord M.G., Adam S.S., Masilamani V. (2018). Management of Menopause Symptoms with Acupuncture: An Umbrella Systematic Review and Meta-Analysis. J. Altern. Complement. Med..

[181] Palma F., Fontanesi F., Facchinetti F., Cagnacci A. (2019). Acupuncture or Phy(F)Itoestrogens vs. (E)Strogen plus Progestin on Menopausal Symptoms. A Randomized Study. Gynecol. Endocrinol..

[182] Melis G.B., Gambacciani M., Cagnacci A., Paoletti A.M., Mais V., Fioretti P. (1988). Effects of the Dopamine Antagonist Veralipride on Hot Flushes and Luteinizing Hormone Secretion in Postmenopausal Women. Obstet. Gynecol..

[183] Cagnacci A., Melis G.B., Paoletti A.M., Soldani R., Fioretti P. (1988). Interaction between Veralipride and the Endogenous Opioid System in the Regulation of Body Temperature in Postmenopausal Women. Life Sci..

[184] Crandall C.J., Mehta J.M., Manson J.E. (2023). Management of Menopausal Symptoms: A Review. JAMA.

[185] Palma F., Fontanesi F., Neri I., Xholli A., Facchinetti F., Cagnacci A. (2020). Blood Pressure and Cardiovascular Risk Factors in Women Treated for Climacteric Symptoms with Acupuncture, Phytoestrogens, or Hormones. Menopause.

[186] Jayasena C.N., Comninos A.N., Stefanopoulou E., Buckley A., Narayanaswamy S., Izzi-Engbeaya C., Abbara A., Ratnasabapathy R., Mogford J., Ng N. (2015). Neurokinin B Administration Induces Hot Flushes in Women. Sci. Rep..

[187] Mittelman-Smith M.A., Williams H., Krajewski-Hall S.J., McMullen N.T., Rance N.E. (2012). Role for Kisspeptin/Neurokinin B/Dynorphin (KNDy) Neurons in Cutaneous Vasodilatation and the Estrogen Modulation of Body Temperature. Proc. Natl. Acad. Sci. USA.

[188] Prague J.K., Roberts R.E., Comninos A.N., Clarke S., Jayasena C.N., Nash Z., Doyle C., Papadopoulou D.A., Bloom S.R., Mohideen P. (2017). Neurokinin 3 Receptor Antagonism as a Novel Treatment for Menopausal Hot Flushes: A Phase 2, Randomised, Double-Blind, Placebo-Controlled Trial. Lancet.

[189] de Oliveira H.M., Diaz C.A.V., Barbosa L.M., Flávio-Reis V.H.P., Zamora F.V., Gonçalves Barbosa Júnior O. (2025). Efficacy and Safety of Fezolinetant and Elinzanetant for Vasomotor Symptoms in Postmenopausal Women: A Systematic Review and Meta-Analysis. Maturitas.

[190] Menegaz de Almeida A., Oliveira P., Lopes L., Leite M., Morbach V., Alves Kelly F., Barros I., Aquino de Moraes F.C., Prevedello A. (2025). Fezolinetant and Elinzanetant Therapy for Menopausal Women Experiencing Vasomotor Symptoms: A Systematic Review and Meta-analysis. Obstet. Gynecol..

[191] Doggrell S.A. (2025). Will Elinzanetant, a Neurokinin Receptor Antagonist, Have a Role in the Treatment of Hot Flashes?. Expert Opin. Pharmacother..

[192] Meczekalski B., Kostrzak A., Unogu C., Bochynska S., Maciejewska-Jeske M., Bala G., Szeliga A. (2025). A New Hope for Woman with Vasomotor Symptoms: Neurokinin B Antagonists. J. Clin. Med..

[193] Nappi R.E., Cagnacci A., Di Carlo C., Genazzani A.D., Villa P., Simoncini T. (2025). Targeting Vasomotor Symptoms with the New Drug Fezolinetant—An Expert Overview. Gynecol. Endocrinol..

[194] Lederman S., Ottery F.D., Cano A., Santoro N., Shapiro M., Stute P., Thurston R.C., English M., Franklin C., Lee M. (2023). Fezolinetant for Treatment of Moderate-to-Severe Vasomotor Symptoms Associated with Menopause (SKYLIGHT 1): A Phase 3 Randomised Controlled Study. Lancet.

[195] Johnson K.A., Martin N., Nappi R.E., Neal-Perry G., Shapiro M., Stute P., Thurston R.C., Wolfman W., English M., Franklin C. (2023). Efficacy and Safety of Fezolinetant in Moderate to Severe Vasomotor Symptoms Associated with Menopause: A Phase 3 RCT. J. Clin. Endocrinol. Metab..

[196] Simon J.A., Anderson R.A., Ballantyne E., Bolognese J., Caetano C., Joffe H., Kerr M., Panay N., Seitz C., Seymore S. (2023). Efficacy and Safety of Elinzanetant, a Selective Neurokinin-1,3 Receptor Antagonist for Vasomotor Symptoms: A Dose-Finding Clinical Trial (SWITCH-1). Menopause.

[197] Pinkerton J.V., Simon J.A., Joffe H., Maki P.M., Nappi R.E., Panay N., Soares C.N., Thurston R.C., Caetano C., Haberland C. (2024). Elinzanetant for the Treatment of Vasomotor Symptoms Associated with Menopause: OASIS 1 and 2 Randomized Clinical Trials. JAMA.

[198] Cardoso F., Parke S., Brennan D.J., Briggs P., Donders G., Panay N., Haseli-Mashhadi N., Block M., Caetano C., Francuski M. (2025). Elinzanetant for Vasomotor Symptoms from Endocrine Therapy for Breast Cancer. N. Engl. J. Med..

[199] European Medicines Agency Veoza Product Information. Revised 21 March 2025. https://www.ema.europa.eu/en/documents/product-information/veoza-epar-product-information_en.pdf.

[200] Shapiro C.M.M., Cano A., Nappi R.E., Santoro N., English M.L., Mancuso S., Morga A., Siddiqui E., Valluri U., Ottery F.D. (2024). Effect of Fezolinetant on Sleep Disturbance and Impairment during Treatment of Vasomotor Symptoms Due to Menopause. Maturitas.

[201] Lieb K., Ahlvers K., Dancker K., Strohbusch S., Reincke M., Feige B., Berger M., Riemann D., Voderholzer U. (2002). Effects of the Neuropeptide Substance P on Sleep, Mood, and Neuroendocrine Measures in Healthy Young Men. Neuropsychopharmacology.

[202] Ratti E., Carpenter D.J., Zamuner S., Fernandes S., Squassante L., Danker-Hopfe H., Archer G., Robertson J., Alexander R., Trist D.G. (2013). Efficacy of Vestipitant, a Neurokinin-1 Receptor Antagonist, in Primary Insomnia. Sleep.

[203] Cano A., Nappi R.E., Santoro N., Stute P., Blogg M., English M.L., Morga A., Scrine L., Siddiqui E., Ottery F.D. (2024). Fezolinetant Impact on Health-Related Quality of Life for Vasomotor Symptoms Due to the Menopause: Pooled Data from SKYLIGHT 1 and SKYLIGHT 2 Randomised Controlled Trials. BJOG.

[204] Haberland C., Barclay M., Lehane A., Whyman S., Gater A., Wikstrom H., Seitz C., Schoof N., Trigg A., Bradley H. (2025). Exit Interviews Examining Changes to Mood and Work/Productivity Impacts Related to Vasomotor Symptoms: Perspectives of Postmenopausal Women Receiving Elinzanetant in Phase III Clinical Trials. Patient.

[205] Panay N., Joffe H., Maki P.M., Nappi R.E., Pinkerton J.V., Simon J.A., Soares C.N., Thurston R.C., Francuski M., Caetano C. (2025). Elinzanetant for the Treatment of Vasomotor Symptoms Associated with Menopause: A Phase 3 Randomized Clinical Trial. JAMA Intern. Med..

[206] Yoshida T., Mio M., Tasaka K. (1992). Cortisol Secretion Induced by Substance P from Bovine Adrenocortical Cells and Its Inhibition by Calmodulin Inhibitors. Biochem. Pharmacol..

[207] Dionysakopoulou C., Lianou L., Boutopoulou B., Giannakopoulou M., Vlachioti E., Koumpagioti D., Bozas E., Matziou V. (2023). The Role of Substance P, Neurokinin A, Neuropeptide Y, and Cortisol in Assessing Neonatal Pain. Neonatal Netw..

